# AI-Enhanced Continuing Professional Development as an Evolving Sociotechnical System: Multimethod Theoretical Framework Development Study

**DOI:** 10.2196/69156

**Published:** 2026-02-12

**Authors:** Vjekoslav Hlede, Sofia Valanci, G Robert D'Antuono, Heather Dow, Ronan O'Beirne, Richard Wiggins

**Affiliations:** 1 Learning Division, American Society of Anesthesiologists, 1061 American Lane, Schaumburg, IL, 60173-4973, United States, 1 7734705592; 2 Office of Learning and Connecting, Royal College of Physicians and Surgeons of Canada, Ottawa, ON, Canada; 3 Office of Continuing Medical Education, NYU Grossman Long Island School of Medicine, New York, NY, United States; 4 Canadian Association of Physical Medicine and Rehabilitation, Kingston, ON, Canada; 5 Division of Continuing Medical Education, Marnix E Heersink School of Medicine, The University of Alabama at Birmingham, Birmingham, AL, United States; 6 Department of Continuing Medical Education, University of Utah, Salt Lake City, UT, United States

**Keywords:** artificial intelligence, AI, AI-enhanced CPD, AI explainability, AI readiness, AI reliability, learning theories, complexity, Actor-Network Theory, black box, continuing professional development

## Abstract

**Background:**

Artificial intelligence (AI) is changing continuing professional development (CPD) in health care and its interactions with the broader health care system. However, current scholarship lacks an integrated theoretical model that explains how AI impacts CPD as a complex sociotechnical system. Existing frameworks usually focus on isolated phenomena, such as ethics, literacy, or learning theory, leaving unaddressed the dynamics of how those phenomena interact in the complex sociotechnical AI-enhanced CPD system, as well as the new roles that AI-empowered patients and society play.

**Objective:**

The objective of this study is to propose a comprehensive, theory-driven framework that provides insight into how AI transforms CPD systems. The goal was to integrate established AI constructs with Complexity Theory (CT) and Actor-Network Theory (ANT) to develop a model that guides practice, research, and policy.

**Methods:**

We conducted a multimethod theory construction. The process started with identifying the AI-enhanced CPD as an established yet evolving phenomenon. Through a structured literature review, the main building blocks of AI-enhanced CPD were identified, as well as the ontological base (CT and ANT). The model was developed through iterative human-led and AI-assisted abductive analysis. The final model was abductively validated on a case study of a national organization pioneering AI use, demonstrating the theoretical model makes sense in practice. All conceptual decisions were reviewed collaboratively by the author group.

**Results:**

The ALEERRT-CA framework is made of 6 pillars: AI literacy, explainability, ethics, readiness, reliability, and learning theories, and 2 theoretical lenses: CT and ANT. CT elucidates macro-level system behaviors in the AI-enhanced CPD system. Those behaviors include emergence, feedback loops, adaptation, and reality made of nested complex systems. ANT explains how localized interactions among human and nonhuman actors shape AI-enhanced CPD. Together, these lenses illustrate how AI redistributes agency, amplifies tensions, and generates emergent learning dynamics within CPD and the broader health care system.

**Conclusions:**

This study presents a novel conceptual model of AI-enhanced CPD as a sociotechnical system. The integration of CT and ANT with AI constructs improves explanatory power of the ALEERRT-CA framework. Educators, program leaders, and policymakers can use the framework as a structured toolset to evaluate AI readiness, design responsible AI-enhanced CPD practices, and plan future empirical research. The framework provides a theoretical lens for observing the rapidly evolving field of AI-enhanced CPD and health care practice.

## Introduction

### AI as a Transformative Sociotechnical Force in Continuing Professional Development

Artificial intelligence (AI) applications are increasingly impacting all aspects of the health care system, from clinical practice (diagnosis, treatment, prevention) to research, communication, administration, and learning [1]. The super-connected, postdigital nature of our society [2] enables AI tools to access vast amounts of data and allows their impacts to spread quickly globally, amplifying AI’s power. AI’s rapid evolution is associated with ethical, legal, social, and professional opportunities and risks for health and continuing professional development (CPD) professionals, patients, and the broader society [3,4]. Therefore, we need to increase our capacity to analyze and improve AI-enhanced CPD and the broader sociotechnical systems with which it is associated [5].

AI tools are gaining transformative powers. They act as a mediating technology that changes how information flows, decisions are made, and how CPD occurs [6]. They are not just new tools improving existing CPD routines; they modify the relationships between clinician-learners, educators, patients, technologies, organizational structures, and regulators.

From an actor-network perspective, AI acts as a nonhuman actor that reorganizes agency, influences interactions, and supports transformations of established professional practices [7]. For example, the introduction of AI into CPD environments contributes to nonlinear, emergent system behavior characteristic of complex sociotechnical adaptive systems, in which small changes in one part of the network can propagate unpredictably across broader educational and clinical contexts [8].

That phenomenon of AI-empowered patients illustrates this transformation. It is reshaping the clinician-patient relationship, influencing decision-making processes, redefining the learning needs of health care professionals, the health system, and the public [9,10]. Those changes can significantly affect patient outcomes.

### Gap, Aim, and Research Question

Despite the increasing importance of AI-enhanced CPD, we lack a framework for examining it as a sociotechnical system. Most published approaches focus on specific issues like ethics, literacy, or digital learning, while leaving out complex relationships between people and technology, as well as the feedback and system dynamics that influence CPD and its role in the broader health care ecosystem.

This study aims to address this by creating an accessible, theory-based framework to analyze how AI changes networks of people and technology, introduces new feedback loops, and transforms CPD systems. Using a multimethod approach based on Borsboom et al [11], we bring together Complexity Theory (CT) and Actor-Network Theory (ANT) to study AI-enhanced CPD. Our primary research question is: How can we best explain AI-enhanced CPD as a sociotechnical system, and what new system-level dynamics and relationships appear when we look at it through both CT and ANT?

This paper makes three contributions. First, it conceptualizes AI-enhanced CPD as a complex sociotechnical system rather than a set of isolated tools. Second, it integrates CT and ANT into a single explanatory framework (ALEERRT-CA). That connects macro-level system dynamics with micro-level human–AI interactions. Third, it provides a reusable analytic tool for educators, organizations, and researchers to evaluate AI readiness, governance, and learning design in CPD contexts.

### Literature Review

AI-enhanced CPD does not emerge in isolation. Our increasingly networked society and health care systems are characterized by increasing interdependence, speed, and systemic complexity [12]. They create a context inseparable from AI-enhanced CPD. In this context, professional learning is no longer bounded by formal educational activities or institutional settings [13]. Instead, AI-enhanced CPD is interwoven with clinical workflows, digital infrastructures, regulatory environments, patient participation, and continuously evolving technologies [14].

This qualitatively changes how CPD functions. Traditional linear, tool-centered, or competency-based models are increasingly insufficient to explain how AI reshapes learning, decision-making, and professional practice across interconnected health care systems [15,16].

This literature review, therefore, approaches AI-enhanced CPD through the dual lenses of complex adaptive systems (CASs) [17,18] and ANT [19]. Rather than treating AI as an isolated educational innovation, we examine AI-enhanced CPD as a dynamic system embedded in a networked society—one in which relationships between human and nonhuman actors, feedback loops, and emergent behaviors are central. This perspective indicates the need for an integrative framework that explains AI’s systemic influence on CPD.

### Changing Complex Systems

CPD acts as an open, adaptive, complex system (CAS) [20,21]. CPD meets established CAS criteria not because of its educational content alone, but because (1) it is embedded within a complex health care system and (2) it tackles complex individual-, team-, and organization-wide learning processes. CPD is structurally connected to clinical workflows, organizational incentives, regulatory regimes, and patient outcomes. CPD is focused on addressing the contextual learning needs of individuals, teams, and organizations and is shaped by emerging technologies and clinical practices. AI adds additional layers of complexity [22]. It provides new variables to the CPD system and connects it more dynamically with the broader health care system.

### Networked Society: A New Context

Initiatives using AI in education have a long history [23]. From teaching machines in the 1920s to expert systems in the 1970s to 1990s, AI tools existed as (semi)isolated centers, relying almost entirely on rules designed by human experts. Now, our society’s super-connected, big-data nature creates a considerably different context. From (semi)isolated centers with limited input of data, AI has become omnipresent and empowered by the global internet of knowledge [24].

Whether we talk about personalized search findings, text editor suggestions, AI-generated meeting minutes, health monitoring apps, 1357 AI-enhanced medical devices recognized by the US Food and Drug Administration [25], learning analytics, or health care quality improvement [26], AI tools are here, tightly embedded in our daily practices.

### There Is Nothing So Practical as a Good Theory

Despite the close relationship between AI, individuals, and society, research on AI in education and CPD has been mostly technocentric and atheoretical [27,28]. Technocentric research may miss the complex interactions between AI, CPD, and broader health care contexts [29].

As we aim to understand how AI impacts CPD activities and the broader sociotechnical health care system, our focus must shift from individual phenomena of AI toward the interaction between AI and various phenomena and activities in the complex context of health care CPD [30]. Theoretical frameworks help us make that shift, allowing us to understand better and manage interactions between various elements of the complex system [31].

Kurt Lewin, one of the pioneers of organizational and social psychology, famously noted, “There is nothing so practical as a good theory” and “the best way to understand something is to try to change it” [32,33]. Guided by those maxims, we propose a theoretical framework to better comprehend and guide the integration of AI and CPD. This is a distinct change within the CPD domain; it creates an opportunity to better understand and improve CPD within the broader sociotechnical context [34,35].

### Why an Integrative Framework Is Needed

Mouloudj et al [36] review of AI adoption in health care has revealed a fragmented landscape of success factors and barriers. Numerous studies describe the importance of AI literacy, explainability, trust, ethical safeguards, organizational readiness, workflow compatibility, social influence, and system reliability. However, these determinants are presented as isolated pillars rather than tightly interconnected components of an AI-enhanced sociotechnical system. In practice, these elements can interact very dynamically. For example, when AI enters a clinical CPD environment, it affects learning design, decision-making processes, roles, relationships, and system behavior.

Since CPD is a CAS inseparable from the complexity of the broader health care system, we need a lens that lets us observe the system and relationships in the system. Focusing on isolated fragments of the system will not provide the needed insight into the system and how AI reshapes it [30].

This creates a gap. We know what forces influence the adoption of AI and that, if properly used, AI can have a transformative positive impact [4]. However, we lack a coherent framework describing how these forces interact across individual, team, organizational, and system levels. AI is being transformed from a tool that supports existing practice into a set of tools that are transforming our sociotechnical system. It reorganizes workflows, amplifies feedback loops, and alters the learning system itself. Therefore, it is becoming increasingly important to have a theoretical lens that can track that transformation.

The ALEERRT-CA framework responds to this need. It brings together 6 foundational AI pillars and situates them within CT and ANT, providing a practical and theoretically grounded way to analyze how AI operates within CPD as a sociotechnical system. By articulating the “anatomy” of AI-enhanced CPD and offering tools to examine both macro-level patterns and micro-level interactions, ALEERRT-CA provides the conceptual infrastructure needed for responsible innovation, rigorous research, and evidence-informed CPD strategy.

## Methods

### Methodology: Theory Construction

This study adopts a theory construction methodology rather than an empirical evaluation design, with the goal of producing an explanatory framework suitable for subsequent empirical testing.

We employed the theory construction methodology outlined by Borsboom et al [11]. This process included the following 5 steps:

1. Identification of Phenomenon: We identified AI-enhanced CPD as a relevant, explainable, and reproducible phenomenon that is rapidly evolving but also stable in terms of its broad and continued impact on CPD [37,38].
2. Drafting Core Principles and Models: VH drafted core principles to explain the phenomenon and created multiple explanatory models using abductive reasoning (explanatory inference). ChatGPT assisted with initial brainstorming and concept review. After concepts were refined, the draft was shared with the author group (2023).
3. Model Development: After multiple additional iterations and contributions from all authors, the final model was developed.
4. Assessment of Explanatory Adequacy: As the fourth step, we assessed the adequacy of the framework and its ability to explain how AI-enhanced CPD can enhance learning.
5. Evaluation of Theoretical Framework: We concluded the process by evaluating the value of the constructed theoretical framework through 2 simulated scenarios and a real-world case study.

### Complexity-Ready Lenses

#### Overview

AI-enhanced CPD occurs in the open, adaptive, complex sociotechnical health care system, where many elements are on the edge of chaos [21,30]. Working with complex, constantly evolving phenomena requires tolerance for ambiguity, a shift of focus from individual phenomena to interactions among multiple phenomena within the system, and the development of unique theoretical tools [30,39]. CT and ANT, 2 complementary theoretical lenses, were used to examine the sociotechnical health care system and the role of AI within it.

#### Selection of the 6 Foundational Pillars

The 6 AI pillars (Literacy, Explainability, Ethics, Readiness, Reliability, and Learning Theories) were selected through an iterative process of pillar identification (Textbox 1). Selection is based on 3 criteria: necessity, nonredundancy, and system relevance. The necessity criterion stipulates that each pillar addresses an important mechanism through which AI influences CPD and the broader health care system. Nonredundancy ensures that no pillar duplicates another pillar’s explanatory function. System relevance requires that each pillar can meaningfully participate in macro-level system dynamics (CT) and micro-level actor interactions (ANT).

Textbox 1. **Construct selection logic (ALEERRT-CA)**

1. Literature frequency and significance: Pillars repeatedly cited across AI, health care, CPD, and education fields
2. Distinct explanatory role: Each pillar explains a unique facet of AI-CPD interaction
3. Alignment with CT and ANT: Must connect to system-level dynamics and actor-level mechanisms
4. Parsimony: Remove overlapping or derivative constructs; retain essential ones

Final Pillars: Literacy, Explainability, Ethics, Readiness, Reliability, and Learning Theories

Constructs frequently mentioned in literature but treated as subsets of broader domains (eg, trust, governance, data quality) were incorporated under more comprehensive theoretical pillars. The final 6 pillars represent the minimal set needed to explain AI’s influence on CPD within a sociotechnical system while maintaining parsimony.

In ALEERRT-CA, learning theories serve a dual role. Epistemologically, they explain how learning occurs across different levels of the system. Functionally, they serve as mechanisms through which AI influences CPD design and outcomes. For this reason, learning theories are treated as both a foundational pillar and a mediating layer within the framework.

### AI-Enhanced Authoring and Analytic Support

**Figure 1.**
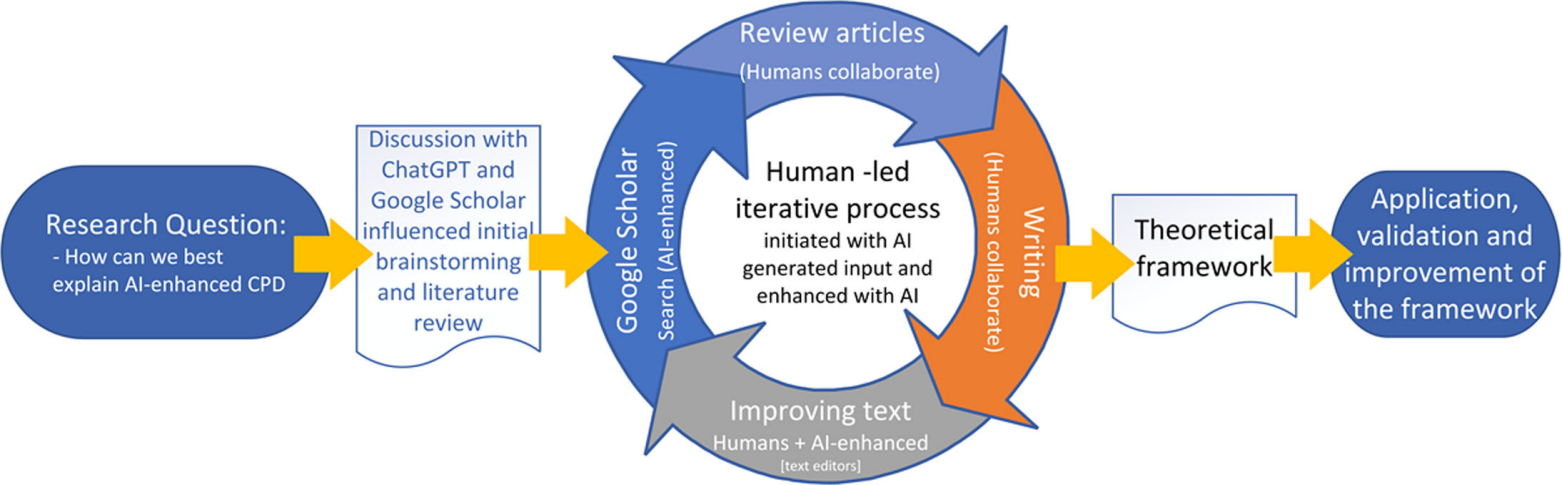
The AI-enhanced writing process of this article. AI: artificial intelligence; CPD: continuing professional development.

All parts of this article—except the practical examples of framework use—were created by human authors with AI support [40]. For example, ChatGPT was used for brainstorming and scenario setting. Google Scholar was used organically to search for literature supporting or expanding the topics discussed. AI-enhanced typing assistants (Grammarly, Microsoft, and Google) were used to improve the text’s clarity, syntax, and concision (Figure 1).

All selected tools come with significant benefits and limitations—most noticeably ChatGPT. For example, ChatGPT output can be biased and lack deep domain-specific insight. ChatGPT cannot make ethical decisions or provide a transparent “thinking process.” Additionally, ChatGPT can hallucinate [26,41]. To address these limitations, we used iterative, collaborative human evaluation and cross-verification with existing literature.

Abductive reasoning was used for framework validation. We tested the framework/hypothesis against real-world case studies.

Due to the complex, emerging nature of the investigated phenomena, inductive and deductive reasoning cannot work well for this task. Therefore, abductive reasoning is the most appropriate approach for this task.

### Ethical Considerations

This manuscript is a conceptual paper. It does not involve human participants, human data, or identifiable personal information. Therefore, per applicable ethical guidelines, including COPE recommendations, ethics committee or institutional review board approval was not required.

## Results

### An Explanatory Framework for AI-Enhanced CPD

This study developed the ALEERRT-CA framework. It is a theory-driven model that provides insight into ways AI can transform CPD as a sociotechnical system. Our multimethod analysis identified 6 central AI pillars and demonstrated how their interactions become clearer when interpreted through CT and ANT. The findings indicated that, in addition to new technological capabilities, AI serves as a transformative agent. It can change relationships, mediating artifacts, and system-level dynamics in CPD and the broader health care system. This study contributes to the conceptual clarity of how AI transforms CPD. It delivers an integrative explanatory model to an under-theorized domain, providing a foundation for future empirical, design-oriented, and policy-focused work in AI-enhanced CPD.

### AI and Learning Theories

Learning theories are an essential building block of AI-enhanced learning environments. For example, Cognitive Load Theory (CLT) and Connectivism can provide valuable insight into using AI to enhance learning activities in different parts of the learning system.

CLT focuses on managing learners’ cognitive load to enhance learning efficiency [42]. The goal is to decrease or eliminate extraneous load (eg, unnecessary examples), adjust the intrinsic load to the learner’s skill level (eg, context appropriate to the skill level), and ensure that the remaining work memory capacity is focused on germane load (ie, cognitive learning processes). Examples of AI-enhanced and CLT-guided interventions are adaptive learning modules, in which AI adjusts difficulty based on learner performance [43,44], and multimedia learning, in which AI optimizes multimedia delivery to optimize cognitive load [45].

Connectivism emphasizes the role of networks and connections in the learning process [46,47]. AI and connectivism may facilitate expanding and managing learning networks in the health care sector [48]. For example, AI can aggregate and curate up-to-date information for health care professionals [49,50] and suggest learning paths based on individual goals and interests.

The examples above illustrate the interaction between learning theories and AI. Learning theories explain how learning happens, and AI can help enhance the learning processes described by learning theories. However, learning theories do not describe the broader framework of how AI, learning practices, and learning theories interact in the broader health care context.

### Framework for Integration of AI and CPD

**Figure 2.**
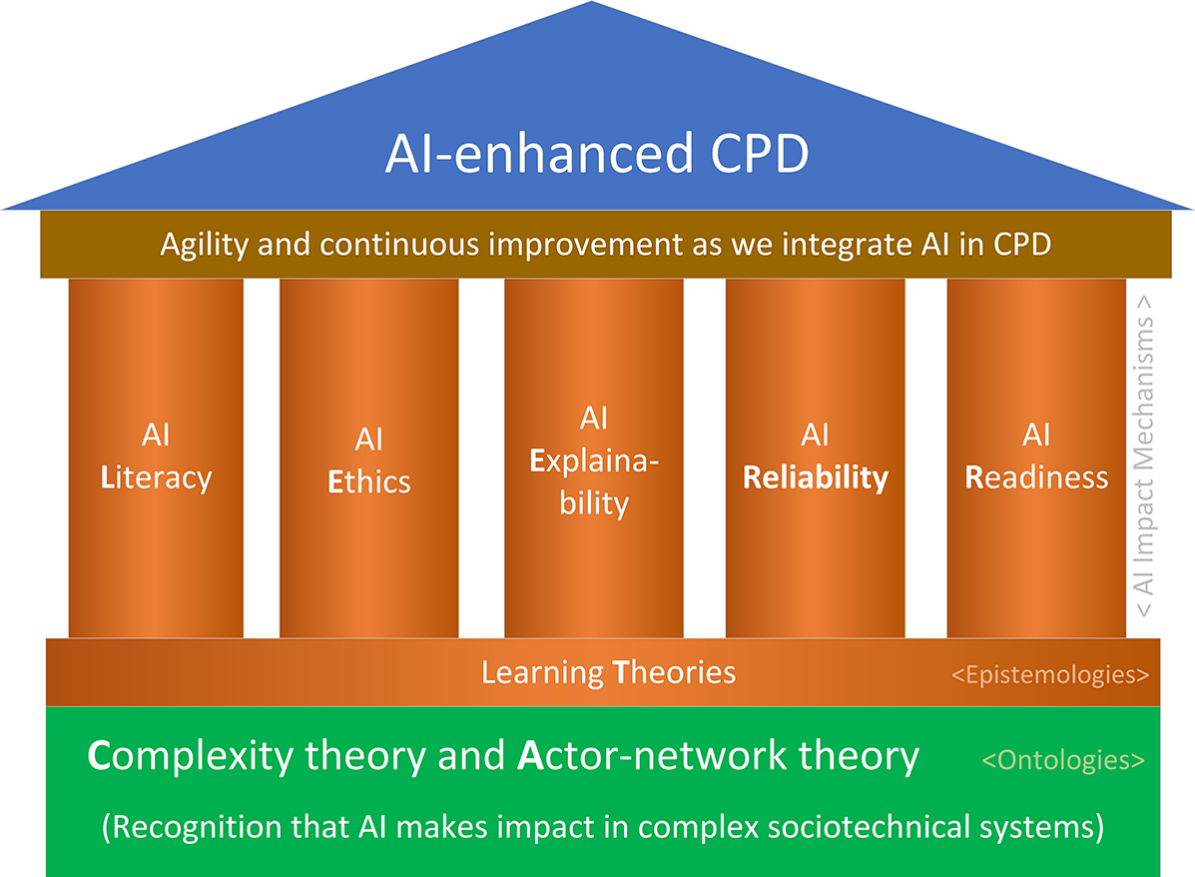
The proposed framework for integrating AI in CPD—ALEERRT-CA is made of the 6 foundational pillars: AI Literacy, Explainability, Ethics, Readiness, Reliability, and Learning Theories, and 2 complementary theoretical lenses to observe system changes associated with AI: Complexity theory and Actor-network theory. AI: artificial intelligence; CPD: continuing professional development.

The complex interaction between AI, learning theories, and CPD practices introduces the need for a framework to comprehend and guide the integration of AI with CPD for health professionals. We propose a framework made of the 6 foundational pillars: AI Literacy, Explainability, Ethics, Readiness, Reliability, and Learning Theories, and 2 complementary theoretical lenses: Complexity theory and Actor-network theory, ALEERRT-CA in short (Figure 2).

AI literacy describes our capacity to understand AI’s basic concepts and principles, such as natural language processing, machine learning, computer vision, and deep data analytics [51]. It also involves the ability to use AI tools and applications in clinical practice, such as decision support systems, diagnostic tools, treatment recommendations, patient monitoring, and health education. AI literacy is essential for health and CPD professionals to leverage AI’s potential for improving quality and efficiency.

**Figure 3.**
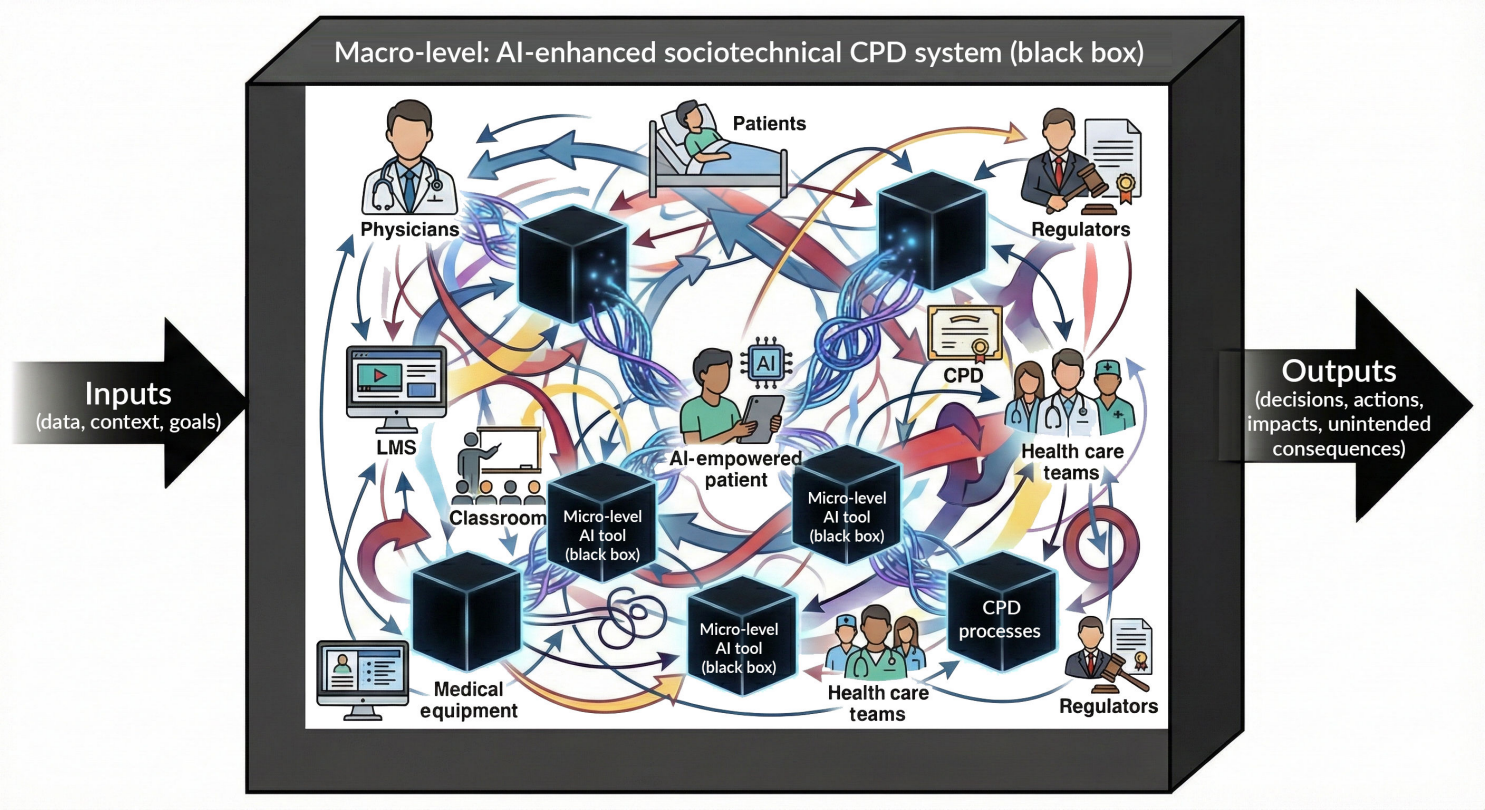
Opening the black box of AI-enhanced CPD. The image presents a conceptual model of an “AI-enhanced sociotechnical CPD system” as a macro-level “black box.” The macro system is a dynamic network of interacting human actors (physicians, patients and their families, and CPD professionals), technical artifacts, and regulatory forces. Crucially, embedded within this chaotic environment are multiple “micro-level AI tools,” presented as opaque black boxes. The diagram illustrates that understanding AI in CPD requires navigating a dual layer of complexity: the lack of explainability in individual AI tools and the unpredictable, emergent dynamics of the broader sociotechnical environment in which they operate. Image created with Google Gemini. AI: artificial intelligence; CPD: continuing professional development; LMS: learning management system.

AI explainability (internal logic of how AI makes decisions) and the explainability of the impact AI makes on learning interventions (relationship between AI actions and broader sociotechnical CPD context) are enablers of AI literacy, readiness, and ethics—understanding how AI-enhanced systems work eases successful implementation of AI [52-54]. However, it seems that trusting that AI is reliable and performs well is more important than having a deep understanding of how AI algorithms work [55]. Very often, we tolerate AI-enhanced solutions as black boxes (Figure 3).

Black boxing is a common process associated with maturation, reliability, and wide acceptance of technology. While the inputs and outputs of the system are known, the user does not understand, or is not even aware of the processes inside the black box. Smartphones are a typical example of a black box [56]. As users, we are experienced in inputting and using smartphone outputs. Yet, the average user is minimally aware of the processes in smartphones and how networked and often AI-enhanced apps in the phone interact with external actors. We focus on the service it delivers to us, not on how it works. We trust it works well.

When failures occur or in attempts to improve the system, we need tools to open black boxes of sociotechnical systems that use AI to make processes visible and ideally understandable to humans [57]. It is an opportunity to examine human and nonhuman actors in the network of relationships between them and how their interactions deliver desired or, in some cases, erroneous outcomes.

AI ethics refers to AI’s ethical, legal, social, and professional implications for health care and CPD practice. It involves protecting patient privacy, obtaining informed consent, ensuring accountability, managing bias, ensuring fairness, protecting intellectual property, promoting transparency, and building trust [58]. AI ethics principles enable health professionals to use AI safely and responsibly.

AI readiness refers to the willingness and preparedness to adopt and implement AI technologies in learning and clinical practice [59]. It involves having a positive attitude and mindset towards AI, being open to learning from and with AI systems, and being able to cope with the changes and challenges that AI brings. AI readiness empowers health professionals to embrace AI as a partner in health care delivery and CPD. This readiness involves properly implementing AI-enhanced, research- and data-driven care by developing mental models, skill sets, and support systems for health care and CPD providers, their teams, and their organizations. Enterprise-wide AI readiness models can be considered to help organize and prioritize the organizational resources for the successful implementation of AI technologies [60-62].

AI reliability is the ability of an AI system to perform consistently and accurately under varying conditions [63]. It is crucial in high-stakes clinical and CPD applications. Yet, it comes with considerable plasticity [64]. AI systems, or humans using AI tools, must be more reliable than humans alone. For example, apps that flag at-risk students or problematic content posted by students may not be as reliable as humans, yet they may reduce the time-consuming task of reading posts and communicating findings, enabling humans to perform much better and faster than they would alone.

Learning theories describe how learning happens and explain where and how AI can improve learning interventions.

CT and ANT are proposed as complementary theoretical lenses, where the CT lens is better suited to deliver a holistic view, while ANT can easily zoom in on a specific part of the system or a specific AI-enhanced app and deliver more actionable insight.

CT explains that our world is made of complex, constantly evolving systems. Those systems are open, and they adapt to changes in the context. Complex systems have emerging properties. Therefore, we cannot understand them simply by analyzing their parts. To analyze AI-enhanced systems, we should not focus solely on AI, but on the system and how the addition of AI is transforming the system.

Complex systems are connected, open, and nested. For example, an individual clinician is a system. Yet she is part of the operating room team—a supersystem. Above that, we have suprasystems such as hospital, national, and global health care systems [65,66]. On all those levels, AI can play a role [67]. Furthermore, AI-related improvement in one system, for example, in the operating room, will stimulate changes in external systems such as CPD, administration, and patient communication.

CT explains the need for multiple learning theories and the interaction between them. Learning theories observe the same phenomena—learning. However, they observe learning in different parts of nested hierarchies of CASs—that is, different contexts [67,68]. The CLT, for example, focuses on individual and learning that happens primarily internally—in a learner’s brain. Connectivism, on the other hand, focuses on learning as a global, social, and technology and artifact-enhanced endeavor. AI can have an impact on all levels of our reality (from individual to global society). Therefore, it is fair to believe that the interaction between AI and established learning theories will be fruitful, allowing us to optimize CPD interventions at every level (individual, team, organization, population, state, and global society) [67].

ANT explains that our reality is shaped through evolving networks of relationships between human and nonhuman actors [69]. ANT posits that nonhuman actors, such as text, digital devices (smartphones or electronic health records), software programs, ideas, organizations, or AI tools, have the agency to shape our reality like humans.

ANT can serve as a magnifier for analyzing micro-level networks of nonhuman and human actors in a specific part of a complex sociotechnical system and at a particular time [69]. It is a good lens to analyze the use of a specific AI-enhanced app or department using AI. CT is better suited to the holistic view of the system [66,70], such as how AI is changing CPD. While the picture created with CT is more inclusive, it is blurry. The macro system’s complex, evolving nature does not allow us to capture all system elements. As a combination, ANT and CT enable us to observe the big picture and, when needed, zoom in and observe a specific part of the AI-enhanced learning health care system.

The framework provides tools that can help us open the black box and observe the “main anatomical structures of AI-enhanced CPD” through both a macro-level (CT) and micro-level (ANT) lenses (Figure 3).

The ALEERRT-CA framework is organized in three conceptual layers. CT and ANT serve as the ontological foundation by defining CPD as a nested, adaptive sociotechnical system made of interacting human and nonhuman actors. Learning theories serve as the epistemological layer, offering assumptions about how knowledge is constructed and how learning can be enhanced. The 6 AI pillars—Literacy, Explainability, Ethics, Readiness, Reliability, and Learning Theories—function as interactional mechanisms that shape how AI influences relationships, behaviors, and emergent patterns within the CPD system. Learning theories simultaneously serve as an AI impact mechanism that enables AI impact and an epistemological lens.

## Discussion

### Reframing AI-Enhanced CPD as a Sociotechnical System

This study presents a novel theoretical lens for analyzing the sociotechnical dynamics of AI-enhanced CPD. It provides insight into how AI reshapes relationships, redistributes agency, and generates emergent learning dynamics across CPD systems. The framework combines 6 AI pillars (AI literacy, explainability, ethics, readiness, reliability, and learning theories) with CT and ANT. The main finding is that complex interactions among system elements are the primary forces shaping AI-enhanced CPD, while isolated AI tools or adoption factors are of secondary importance. Due to its complex nature, AI-enhanced CPD often acts as a black box. The framework strengthens our capacity to analyze the complex sociotechnical nature of AI-enhanced CPD.

### Adequacy of the Framework

The proposed framework provides a checklist of 6 building blocks of AI implementation and 2 theoretical lenses to observe system changes associated with AI. It is a tool set that can help us plan AI implementation and, when needed, open black boxes of AI-enhanced CPD. Therefore, it addresses the criteria of practical application and simplicity (parsimony) [71]. CT and ANT, as macro-level and micro-level theories, allow us to observe the system and large-scale structures (CT), and, when needed, focus on the interaction between a small network of human and nonhuman actors (ANT). The proper theoretical lens for a proper task model aligns with the principle of parsimony.

The framework is rooted in 2 established theories (CT and ANT) and the literature on AI implementation, which enhances its external consistency and ensures it fits with the broader theoretical landscape of AI in CPD. It appears to provide a good balance of practicality, simplicity, and theoretical robustness, suggesting it can effectively explain how successful AI-enhanced improvement of CPD can occur.

### Value of the Constructed Theoretical Framework

The ALEERRT-CA allows us to open the black boxes of AI-enhanced CPD. The black boxes exist on at least 2 levels. The first level is AI algorithms. That level is not addressed in this paper. On the level above, where this framework is focused, we have AI-enhanced sociotechnical systems and need to understand hidden, complex interactions between AI, technical, and social elements that influence learning health system performance and outcomes. The framework describes the main parts—the anatomy—of the AI-enhanced CPD and 2 theoretical lenses used to analyze them.

AI implementation-focused guides, such as Rashidi Your AI Survival Guide [72], highlight many of the conditions necessary for successful AI adoption, including literacy, workflow redesign, trust, governance, readiness, and interprofessional collaboration. These themes map closely onto the ALEERRT-CA pillars. The proposed framework builds on that. ALEERRT-CA extends beyond operational guidance.

Whereas leadership manuals explain what organizational leaders must pay attention to, ALEERRT-CA explains how these elements interact within a complex adaptive learning ecosystem and why AI reshapes roles, routines, and system behaviors. By embedding AI constructs within CT and ANT, the framework provides a level of explanatory depth that practice-oriented texts do not have. The framework explains the dynamics through which AI mediates relationships, generates tensions, and produces emergent patterns in CPD. In this way, ALEERRT-CA transforms a collection of best practices into a coherent, theory-driven model capable of guiding both empirical research and strategic system design.

### Interaction Between Pillars and Theoretical Lenses

**Figure 4.**
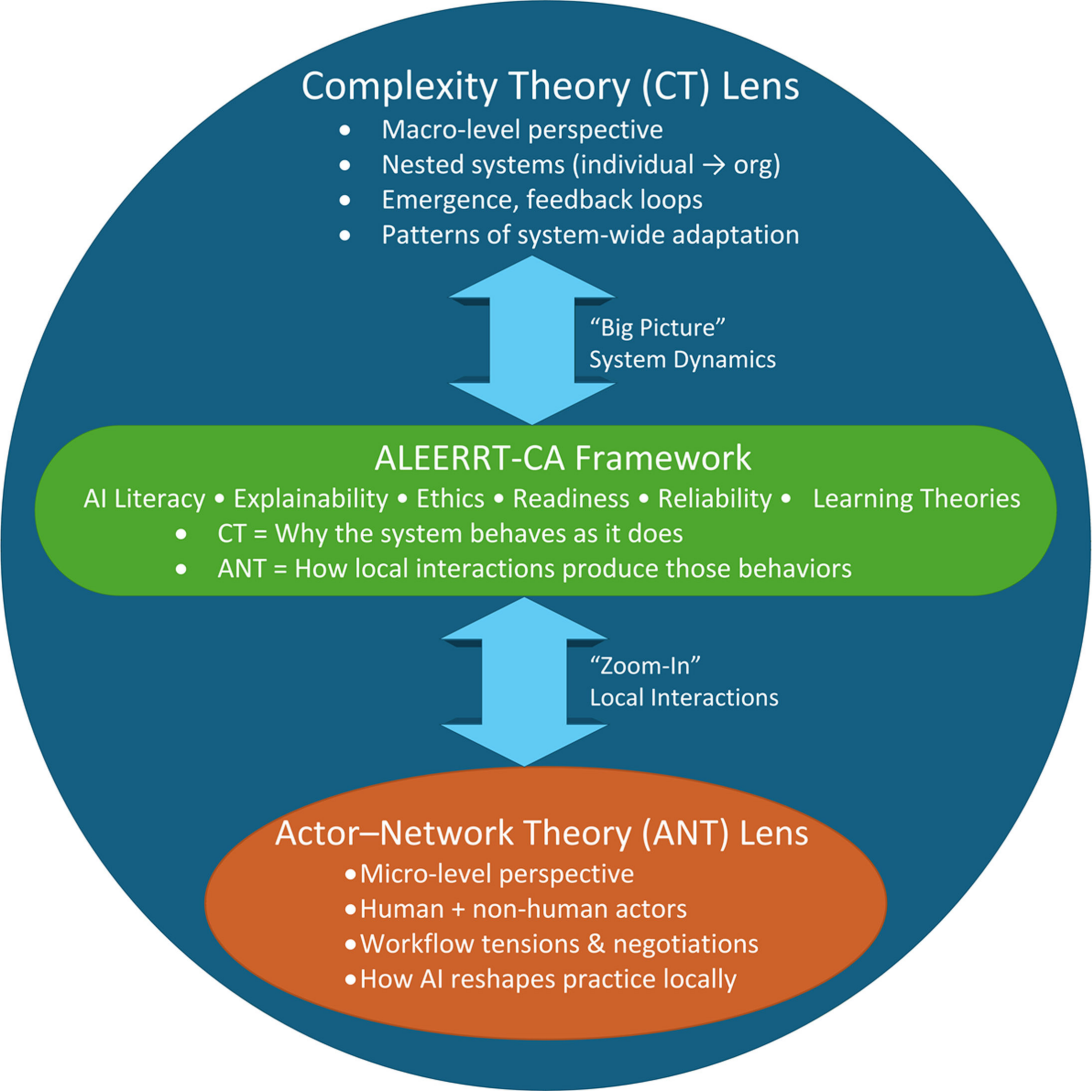
Complexity Theory (CT) and Actor-Network Theory (ANT) serve as complementary analytical lenses within the ALEERRT-CA framework. CT provides a macro-level view of continuing professional development (CPD). Through that lens, CPD is seen as a nested, adaptive system shaped by emergence, feedback loops, and system-wide interactions. ANT, on the other hand, provides a micro-level view. Through that lens, CPD is seen as a network of human and nonhuman actors (clinicians, educators, learners, AI tools, platforms, and policies). We can see how they interact and negotiate new practices. Together, CT and ANT enable ALEERRT-CA to explain both broad system transformation and the specific mechanisms through which AI reshapes CPD.

The 6 pillars of ALEERRT-CA demonstrate distinct yet interdependent explanatory functions when analyzed through ANT and CT (Figure 4).

For example, ANT shows that explainability is not a feature strictly associated with AI algorithms. Reality is more complex. Explainability is negotiated across a network of users, artifacts, interfaces, and organizational structures. Therefore, explainability is relational, contextual, and dynamic. What counts as “a good explanation” varies with the actor making the assessment, purpose, location within the system, and level of risk.

ANT informs AI ethics by showcasing how agency, accountability, and power are redistributed when nonhuman actors participate in decisions. System-wide ethical outcomes are created through interactions among designers, clinicians, learners, data, models, platforms, policies, and workflows. ANT enables CPD leaders to evaluate not only whether AI is ethical, but how ethical behavior is produced, constrained, or undermined by the network.

Yang et al [73] provide a practical example. Explainability and perceived reliability during peer reviews are shaped by the social “advice-taking phenomenon.” Physicians who openly use AI tools during decision-making usually receive lower competence ratings, despite shared belief that AI is helpful. Positioning AI as a verification tool only moderately reduces that negative effect. The probable reason is that we have a significant reconfiguration of the actor network. Epistemic authority, agency, and accountability are redistributed and reshaped by AI. That change reduces trust and perceived reliability, despite improved performance.

As illustrated in Table 1, ANT enables micro-level analytic tracing of how AI reshapes agency, accountability, and epistemic authority through human–nonhuman associations. CT complements this by explaining how localized interactions among these actors influence emergent system trajectories across nested sociotechnical strata. Together, the dual-lens positioning of ALEERRT-CA reframes AI-enhanced CPD not as a linear implementation challenge but as a dynamic process of relational negotiation and systemic adaptation. This dual-theoretical integration provides explanatory depth and design utility, capturing both mechanism and emergence.

**Table 1.** Theoretical interaction of ALEERRT-CA pillars with Actor-Network Theory and Complexity Theory.

ALEERRT-CA pillar	ANT^a^ contribution, a micro-level analytical mechanism	CT^b^ contribution, a macro-level system mechanism	Theoretical implication
AI^c^ literacy	Positions literacy as a prerequisite for relational/contextual agency. Actors with greater literacy have disproportionate influence over problem definition, meaning-making, and artifact interpretation.	Literacy accelerates or constrains system adaptation; uneven literacy becomes a systemic bottleneck spreading nonlinearly across teams and institutions.	AI literacy shapes epistemic power and mediates how knowledge circulates within sociotechnical networks.
Explainability	ANT frames explainability as an outcome of interaction between various human and nonhuman actors and ideas; what is “understandable” is rooted in actor identity, status/position in the networks, network structures, and interpretive frames.	Changes in the level of explainability can amplify or suppress feedback loops, affecting trust, adoption, and the rate of transformation. Explainability is needed at multiple levels: from the work of AI algorithms, individual-focused AI-enhanced CPD^d^, team- and organization-focused learning, and integration between learning and clinical practice.	Explainability is relational, negotiated, and context-dependent rather than a static technical property. Explainability of processes (eg, AI algorithms) on one level influences explainability on other levels (eg, clinical practice)
Ethics	Shows how responsibility and agency are redistributed when nonhuman actors coproduce decisions and outcomes.	Ethical disruptions propagate via feedback loops, enabling amplification of harms or benefits at scale. Ethics is important on all levels: algorithm, individual, team, organization, society	Ethics becomes a governance outcome of network configuration, not solely adherence to level-specific principles.
Readiness	Readiness depends on network alignment. Misaligned goals, competencies, and affordances produce friction and resistance.	Initial conditions and small deviations shape emergent trajectories (“sensitivity to initial states”). Readiness is multilayered. At different levels of system aggregation (individual, team, organization, society), we can observe considerable differences in readiness.	Readiness is dynamic and coevolving, requiring continual recalibration across nested systems.
Reliability	Reliability emerges from situated interaction between human and nonhuman actors; failure modes are relational.	Failures or successes can trigger rapid changes in trust in AI-enhanced systems. That can impact behavior across the system(s).	Reliability is contingent and contextual, negotiated by the system (and between systems) rather than guaranteed by the artifact.
Learning theories	ANT^a^ reveals how AI acquires pedagogical agency (content generation, evaluation, orchestration) and shapes relationships in a network of actors.	CT^b^ positions AI-enhanced learning as emergent across individual, social, and organizational strata.	CPD^d^ must align AI-enhanced learning design with both cognitive and sociotechnical learning dynamics. AI can enhance individual, team, and system learning.

a ANT: Actor-Network Theory.

b CT: Complexity Theory.

c AI: artificial intelligence.

d CPD: continuing professional development.

To illustrate the value of the ALEERRT-CA framework, we offer an explanatory case.

### Explanatory Case: The American Society of Anesthesiologists BeaconBot and the Emerging AI-Enhanced CPD Ecosystem

The American Society of Anesthesiologists (ASA) is coproducing an AI-enhanced learning ecosystem that tackles clinical practice, quality improvement, education, research dissemination, and workforce development. A central AI-enhanced component is ASA BeaconBot, a generative AI knowledge assistant available to ASA members [74]. BeaconBot synthesizes information from ASA’s public materials, member-only resources, *Anesthesiology® journal*, committee documents, and ASA Standards, Guidelines, and Statements. BeaconBot functions as a real-time mediator of organizational knowledge, supporting clinicians, educators, and staff in accessing evidence, policies, and practice recommendations more efficiently. It serves as a librarian, quickly providing answers to complicated questions with citations and links to sources that informed the answers.

In parallel, the Anesthesia Quality Institute is advancing a multistage integration strategy linking (1) Epic electronic health record data, (2) the National Anesthesia Clinical Outcomes Registry [75], and (3) ASA’s association management system. Public descriptions of this work emphasize using high-volume clinical data to improve benchmarking and quality improvement across anesthesia practices.

A long-term, aspirational goal, currently conceptual rather than operational, is to explore whether abstracted, nonpatient-identified insights about clinicians’ practice patterns (eg, procedural focus, strengths, and learning needs) could be generated in the future. At present, legal, regulatory, and data privacy constraints limit such use.

These insights could eventually inform ASA’s learning management system and learning experience platform, enabling AI-supported personalized CPD recommendations aligned with actual clinical practice [76].

ASA operates across multiple educational layers: individual clinicians and residents, perioperative teams, organizational purchasers of CPD, national public education and legislative advocacy, and international patient-safety leadership. Across these layers, AI is already entangled with everyday practice: faculty and members use AI tools for literature synthesis, communication, research workflows, and meeting documentation. Many committee processes and reports are generated or refined with AI-assisted writing tools, and several e-learning materials, including SCORM (Shareable Content Object Reference Model) e-learning modules, are produced with AI-supported content development [77].

Internally, ASA is implementing an empowered product team model [78]. The traditional hierarchical and often siloed, feature-focused waterfall project management practices are being replaced with an agile continuous discovery model built around the active involvement of all stakeholders [79].

The empowered product teams model aligns with both CT and ANT, as it operates as an adaptive, feedback-driven hub capable of responding to emergent interactions among human and nonhuman actors. In contrast, waterfall models assume stability, linearity, and fixed requirements—assumptions that are poorly aligned with the aim of innovation in AI-enhanced CPD systems.

Viewed through the ALEERRT-CA framework, this evolving ecosystem illustrates:

- Literacy: Heterogeneous AI literacy across clinicians, faculty, learners, and staff creates both capability gaps and opportunities for targeted support.
- Explainability: BeaconBot’s responses and any future recommendation logic require transparent reasoning pathways for clinicians, educators, and regulators. In addition to how recommendations are created, we want to know how they help clinicians improve their practice.
- Ethics: Registry-to-learning integration raises governance questions around data use, privacy, and boundaries between performance data and educational personalization.
- Readiness: Organizational readiness fluctuates across departments as product teams, educators, IT, and leadership adapt to new workflows and AI-mediated artifacts.
- Reliability: Perceived reliability depends on both technical accuracy and user trust, particularly when AI synthesizes standards or summarizes clinical knowledge.
- Learning theories: Learning shifts from content-centric modules toward system-wide, data-driven adaptive learning, where clinical practice informs CPD and CPD feeds back into practice.

From a CT perspective, ASA’s system shows nonlinear interactions: small changes (eg, improved data quality, AI-generated insights, or revised governance policies) can propagate across clinical, educational, and organizational layers. From an ANT lens, AI agents (BeaconBot, analytics engines, transcription models, generative AI) operate as nonhuman actors that mediate relationships among clinicians, educators, committees, systems, and CPD infrastructures. Those actions empower CPD to act as a learning health ecosystem, characterized by evolving feedback loops, distributed agency, and emergent patterns of adaptation.

While results are promising, strong tensions around data governance, ethics, explainability, change fatigue, and the need for AI literacy among clinicians, faculty, and staff impede progress.

This case illustrates how AI-enhanced CPD cannot be understood as a discrete educational intervention but must be analyzed as a system-level transformation involving data flows, governance, professional identity, and learning infrastructure—precisely the analytic space addressed by ALEERRT-CA. Thus, it is equally beneficial for organizations and CPD professionals.

As a relatively simple but inclusive and complexity-ready model, it can serve as a toolset that will help us with the integration of AI in CPD. It can support interdisciplinary collaboration between medical, engineering, social, ethical, and legal domains and help us shape human-centric AI-enhanced CPD.

As with physical toolsets, it is not necessary to use all tools in the box for every situation. Some analytic tasks require only a subset of the framework’s components. The framework is also extensible. While CT and ANT are well-suited for explaining macro-level system dynamics and micro-level sociotechnical interactions in AI-enhanced CPD, other theoretically compatible lenses could be incorporated if they better match a specific analytic need or reflect the expertise of a given research team.

### Limitations

The complex, constantly evolving, and rapid nature of AI in our society requires an agile, continuous-improvement mindset. Therefore, we propose this framework as a work in progress and a position on the direction we, as the health care CPD community empowered with AI tools, may take. The framework does not claim completeness or finality but is intended as a theoretically grounded starting point for iterative refinement through empirical studies.

The iterative nature of the framework allows us to improve both our use of it and the framework itself. Similarly, repeated empirical validation will help confirm its value.

### Conclusion

Integration of AI into health care CPD is reshaping a complex system, demanding a deeper understanding and strategic application of available resources.

This work introduces ALEERRT-CA, a theory-driven framework that provides a lens for observing dynamic interactions in AI-enhanced CPD. The framework explains how AI reorganizes relationships, redistributes agency, and generates emergent learning across health care and CPD systems.

ALEERRT-CA can serve as an analytic tool for practice. Educators and organizational leaders can use the tool to assess AI-enhanced learning at multiple levels, from individual learners to organizations and broader health care systems. The paired macro and micro lenses support both strategic “big picture planning” and focused analysis of local AI-enhanced CPD initiatives.

### Lessons for Practice

As AI becomes increasingly embedded in healthcare education and practice, CPD professionals are faced with practical decisions about how to design, implement, and govern AI-enhanced learning. The following lessons for practice distill key insights from this study:

- AI-enhanced tools have become part of our daily lives. It is not whether we use AI, but how effectively we use it to enhance our CPD practices.
- AI makes an impact on complex sociotechnical systems. Therefore, AI-enhanced CPD interventions should be designed to foster beneficial interactions between AI tools, health care professionals, and the broader health care system.
- Theoretical tools, such as ALEERRT-CA, can increase our ability to open the black boxes of AI-enhanced CPD sociotechnical systems and understand and improve how AI is used in CPD, ultimately improving the impact of CPD.

## Abbreviations

AI: artificial intelligence
ALEERRT-CA: AI Literacy, Explainability, Ethics, Readiness, Reliability, and learning Theories-Complexity theory and Actor-network theory
ANT: Actor-Network Theory
ASA: American Society of Anesthesiologists
CAS: complex adaptive system
CLT: Cognitive Load Theory
CPD: continuing professional development
CT: Complexity Theory
